# Experimental and Mathematical Modelling of Factors Influencing Carbon Dioxide Absorption into the Aqueous Solution of Monoethanolamine and 1-Butyl-3-methylimidazolium Dibutylphosphate Using Response Surface Methodology (RSM)

**DOI:** 10.3390/molecules27061779

**Published:** 2022-03-08

**Authors:** Fatin Nor Arissa Azhar, Mohd Faisal Taha, Siti Musliha Mat Ghani, Muhammad Syafiq Hazwan Ruslan, Noor Mona Md Yunus

**Affiliations:** 1 Fundamental and Applied Science Department, Universiti Teknologi PETRONAS, Seri Iskandar 32610, Malaysia; fatin.nor_24538@utp.edu.my; 2 Centre of Research in Ionic Liquids, Universiti Teknologi PETRONAS, Seri Iskandar 32610, Malaysia; siti_17007548@utp.edu.my (S.M.M.G.); mona.yunus@utp.edu.my (N.M.M.Y.); 3 School of Chemical Engineering, College of Engineering, Universiti Teknologi MARA, Shah Alam 40450, Malaysia; syafiqhazwan@uitm.edu.my

**Keywords:** carbon dioxide, ionic liquid, absorption

## Abstract

This paper investigated the solubility of carbon dioxide (CO_2_) in an aqueous solution of monoethanolamine (MEA) and 1-butyl-3-methylimidazolium dibutylphosphate ((BMIM)(DBP)) ionic liquid (IL) hybrid solvents. Aqueous solutions of MEA-(BMIM)(DBP) hybrid solvents containing different concentrations of (BMIM)(DBP) were prepared to exploit the amine’s reactive nature, combined with the IL’s non-volatile nature for CO_2_ absorption. Response surface methodology (RSM) based on central composite design (CCD) was used to design the CO_2_ solubility experiments and to investigate the effects of three independent factors on the solubility of CO_2_ in the aqueous MEA-(BMIM)(DBP) hybrid solvent. The three independent factors were the concentration of (BMIM)(DBP) (0–20 wt.%), temperature (30 °C–60 °C) and pressure of CO_2_ (2–30 bar). The experimental data were fitted to a quadratic model with a coefficient of determination (*R*^2^) value of 0.9791. The accuracy of the developed model was confirmed through additional experiments where the experimental values were found to be within the 95% confidence interval. From the RSM-generated model, the optimum conditions for CO_2_ absorption in aqueous 30 wt% MEA-(BMIM)(DBP) were 20 wt% of (BMIM)(DBP), a temperature of 41.1 °C and a pressure of 30 bar.

## 1. Introduction

The increasing amount of CO_2_ in the atmosphere has critically impacted the environment and human health [1]. CO_2_ emissions mostly come from fossil fuel combustion in the energy sector, natural gas streams and other industrial processes [2]. Fossil fuel power plants are usually integrated with CO_2_ capture technologies based on solvents’ chemical absorption. Currently, the commercial solvents that are being used as chemical adsorbents in CO_2_ capture technologies are alkanolamines such as monoethanolamine (MEA), diethanolamine (DEA) and N-methyldiethanolamine (MDEA). However, the use of these alkanolamines presents several disadvantages such as corrosion, thermal and oxidative degradation, leading to the loss of absorbents, limited CO_2_ loading and high regeneration energy and cost [3,4].

ILs possessing significant characteristics, such as negligible volatility, high thermal stability and tunable physicochemical properties, have been demonstrated to effectively absorb CO_2_ [5]. The combination of more stable ILs with reactive alkanolamines, known as hybrid solvents, has demonstrated better CO_2_ absorption and fewer absorbent losses as compared to alkanolamines alone [6]. In addition, ILs were reported to have less than one-third of the heat capacity of water in a wide range of temperatures, which could significantly reduce the regeneration energy, which in turn reduces the operational cost [7,8]. Mixing alkanolamines with ILs would also be advantageous to overcome the problems associated with the high viscosity and high cost of ILs for the industrial application of CO_2_ absorption. Furthermore, considering the widespread use of alkanolamine CO_2_ capture technology, the addition of small quantities of IL into aqueous commercial alkanolamines would not create significant changes in the existing process designs [9].

In this work, hybrid solvents comprising a fluorine-free IL, i.e., (BMIM)(DBP), and aqueous 30 wt% MEA were studied for the removal of CO_2_. The MEA concentration was kept constant at 30 wt% as this concentration of MEA is widely used in the current CO_2_ absorption technology [10]. The IL (BMIM)(DBP) was selected due to its miscibility with water and its CO_2_ capture capacity, which is comparable to 1-butyl-3-methylimidazolium bis(trifluoromethylsulfonyl)imide ((BMIM)(Tf_2_N)). It was suggested that the longer alkyl chain in the dibutylphosphate anion creates a larger free volume to accommodate more CO_2_ molecules [11,12]. The relationship between the physical solubility of gases in other ILs and the free volume of ILs was intensively discussed in Hu et al. and Shannon et al. [13,14]. The solubility of CO_2_ in this aqueous MEA-(BMIM)(DBP) was investigated at different (BMIM)(DBP) concentrations, temperatures, and pressures according to the experimental design generated by means of the response surface methodology (RSM) using central composite design (CCD). The RSM was applied to identify the optimum level of factors that generate high efficiency of CO_2_ absorption.

RSM is a collection of mathematical and statistical techniques that are useful for modeling and analysis in applications where a response of interest is influenced by several factors. RSM can be used to optimize processes, evaluate simultaneous effects of factors and predict the response of a process to new factors and conditions [15]. CCD is one of the design strategies in RSM and CCD has been used widely due to its advantage of using a small number of experimental runs. Morero et al. reported work on applying RSM to evaluate parameters in upgrading biogas [16]. Pashaei et al. analyzed the optimization of CO_2_ absorption in piperazine solution using RSM-CCD [17].

The main objectives of this study are to investigate three independent factors (the concentration of (BMIM)(DBP), temperature and the pressure of CO_2_) affecting CO_2_ absorption in aqueous 30 wt% MEA-(BMIM)(DBP) hybrid solvent and to predict the optimum conditions that would lead to high CO_2_ absorption.

## 2. Materials and Methods

### 2.1. Materials

1-butyl-3-methylimidazolium dibutylphosphate ((BMIM)(DBP)) and monoethanolamine (MEA) were purchased from Merck. Carbon dioxide gas with a purity of 99.99% was purchased from Linde.

### 2.2. Density and Viscosity Measurement

The density of all aqueous MEA-(BMIM)(DBP) samples was measured using a Stabinger density viscosity meter (Anton Paar SVM3000, Anton Paar, Graz, Austria) with a precision of ±0.0001 g/cm^3^. All measurements of the density were performed at 30 °C, 45 °C and 60 °C. The accuracy of the Stabinger viscometer was verified with certified reference fluids N14 and N44, which were obtained from Cannon Instrument Company (State College, PA, USA). The uncertainties of the instrument were ±0.01 °C and ±0.0005 g/cm^3^ for temperature and density, respectively. The viscosity of all aqueous MEA-(BMIM)(DBP) samples was measured using a TA Instrument DHR-1 rheometer at 30 °C, 45 °C and 60 °C. The instrument was calibrated with certified reference fluid S600, which was obtained from Cannon Instrument Company (State College, PA, USA). The uncertainty of the temperature was ±0.1 °C and the expanded uncertainty of the dynamic viscosity was ±0.4%.

### 2.3. Heat Capacity Measurement

The measurement of heat capacity was performed using a heat flow differential scanning calorimeter (DSC) (model DSC1, Mettler Toledo, Columbus, OH, USA). For each measurement, 50–60 mg of the sample, encapsulated in an aluminum pan, was used. The obtained differential heat flow curve of the sample was compared with that of standard sapphire (with both curves blank-corrected). The purge gas used was nitrogen (purity > 99.9995%) at a flow rate of 50 mL/min. Calibration of the DSC using indium as the calibrant was also conducted to ensure the accuracy of the measurements. The uncertainties of the instrument were ±0.6 °C and ±2 J/g for temperature and heat flow, respectively.

### 2.4. Solubility of CO_2_

**Figure 1:**
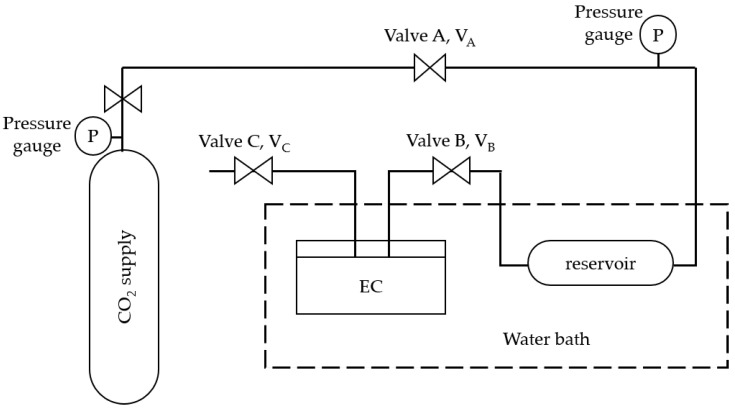
Schematic diagram of CO_2_ absorption apparatus.

The CO_2_ solubility in the sample was carried out based on the isochoric saturation method [18,19]. A high-pressure equilibrium cell (EC) with a capacity of 15 mL, made of stainless steel, was used to carry out the experiments. The equilibrium cell was attached to a pressure gauge and temperature controller having ranges from 0 to 40 bar and room temperature to 80 °C, respectively. A schematic of the experimental setup is shown in Figure 1.

During the experiment, a known quantity of sample (1.0 to 1.5 g) was loaded into the equilibrium cell (EC). The EC was degassed by means of a vacuum pump and the desired temperature inside the EC was maintained using a water bath. CO_2_ gas was then introduced into the reservoir of a known volume from Valve A (V_A_) to Valve B(V_B_) and brought to a constant temperature. The initial number of moles of CO_2_ was calculated using Equation (1).

(1) $$n_{CO_{2}}^{i}=\frac{P_{i}V_{i}}{Z_{CO_{2}}^{i}RT_{i}}$$

where $n_{CO_{2}}^{i}$ is the initial number of moles of CO_2_ charged into EC, $P_{i}$ is the initial pressure (atm), $T_{i}$ is the initial temperature (K), $V_{i}$ is the volume of the CO_2_ absorption system from V_A_ to V_B_ (L), $z_{CO_{2}}^{i}$ is the compressibility factor at initial temperature and pressure conditions (calculated using the Peng–Robinson equation of state) and *R* is the universal gas constant. CO_2_ was then introduced into EC by opening V_B_. As the absorption of CO_2_ in the sample starts, the pressure inside the cell decreases continually. The pressure in the system was recorded in 1 min intervals, as the whole system was given sufficient time to reach the equilibrium. The experiment was stopped when the pressure remained constant for 30 min. The equilibrium time duration varied between 90 and 120 min. In the equilibrium conditions, the moles of CO_2_ left in the cell were calculated using Equation (2).

(2) $$n_{CO_{2}}^{eq}=\frac{P_{eq}(V_{total}-V_{s})}{Z_{CO_{2}}^{f}RT_{eq}}$$

where $n_{CO_{2}}^{eq}$ is the number of moles of CO_2_ left in the system at equilibrium (mole), $P_{eq}$ is the pressure at equilibrium (atm), $T_{eq}$ is the temperature at equilibrium (K), $V_{total}$ is the volume (L) of the CO_2_ absorption system from V_A_ to Valve C (V_C_), $V_{s\;}$ is the volume of sample (L) and $Z_{CO_{2}}^{i}$ is the compressibility factor at equilibrium temperature and pressure conditions (calculated using the Peng–Robinson equation of state). The number of moles of CO_2_ absorbed ($n_{CO_{2}}^{abs})$ by the sample is given by Equation (3).

(3) $$n_{CO_{2}}^{abs}=n_{CO_{2}}^{eq}-n_{CO_{2}}^{i}$$

where $n_{CO_{2}}^{eq}$ is the number of moles of CO_2_ left in the system at equilibrium and $n_{CO_{2}}^{i}$ is the initial number of moles of CO_2_ charged into the EC. Meanwhile, the solubility of CO_2_ expressed in mole fraction ($X_{CO_{2}}$) was calculated according to Equation (4).

(4) $$X_{CO_{2}}=\frac{n_{CO_{2}}^{abs}}{n_{CO_{2}}^{abs}+n_{s}}$$

where $n_{CO_{2}}^{abs}$ is the number of moles of CO_2_ absorbed by the sample and $n_{s}$ is the number of moles in the sample. The apparatus setup and procedure used in this work were verified using the aqueous 30 wt% MEA. The absolute relative deviation for the experimental CO_2_ solubility value of this work and the literature value [20] was less than 5%. The instrumental uncertainties in temperature and pressure were ±0.1 °C and ±0.1 bar, respectively, whereas the relative standard uncertainty in the CO_2_ solubility in the mole fraction, estimated from the standard deviation of the measurements, was ±4%.

### 2.5. CO_2_ Solubility in the Aqueous MEA-(BMIM)(DBP) Using RSM-CCD

The design of experiments (DOE) was developed using Design-Expert software version 12 based on the Face-Centered CCD (FCCCD) with CO_2_ absorption as the dependent response. The factors of the experimental design, i.e., IL concentration (wt%), temperature (°C) and pressure (bar), were selected. The FCCCD was selected according to three levels and three variable concepts. A total of 16 unique runs with 4 mid-point replication was proposed to give a total of 18 experimental runs. The three independent variables were prescribed into three levels (low, middle and high) and coded values (−1, 0, +1). CCD was selected because the design includes a repetition of center points that are used to calculate the experimental error, which provides more reliable data. The experimental results were fitted into a regression model equation for modeling purposes.

In this study, the independent variables for the (BMIM)(DBP) concentration, temperature and pressure were denoted, respectively, by *X*_A_, *X*_B_ and *X*_C_. The range and levels of the processing parameters involved are shown in Table 1.

## 3. Results and Discussion

### 3.1. Density and Viscosity

**Figure 2:**
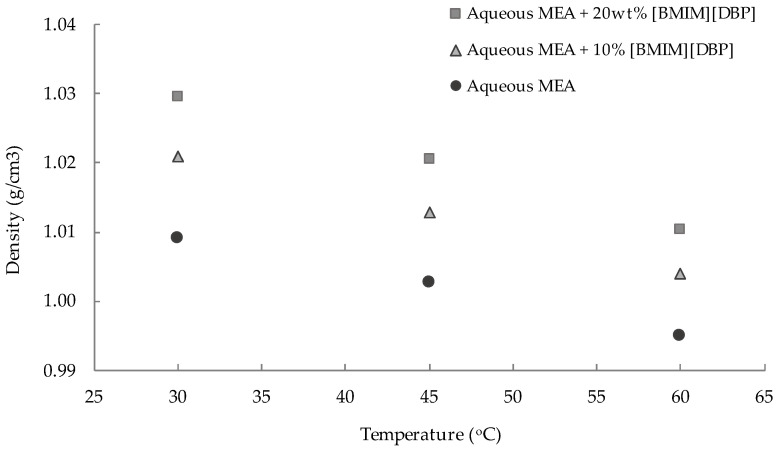
Density of aqueous 30 wt% MEA-(BMIM)(DBP) hybrid solvents containing different (BMIM)(DBP) concentrations at different temperatures (30 °C, 45 °C and 60 °C) and at pressure of 1 atm.

Figure 2 shows the significant effect of IL concentration and temperature towards the density of the aqueous MEA-(BMIM)(DBP) solvent. As expected, the density of the hybrid solvent increased with an increase in the (BMIM)(DBP) concentration. This is due to the density of (BMIM)(DBP) (1.04 g/cm^3^) [21], which is higher than that of MEA (1.015 g/cm^3^) [22]. The density of the hybrid solvent decreased with increasing of the temperature. This could be due to the fact that the increase in temperature weakened the molecular interactions between the molecules; therefore, the density of ILs or amine-IL hybrid solvents decreased [23,24].

**Figure 3:**
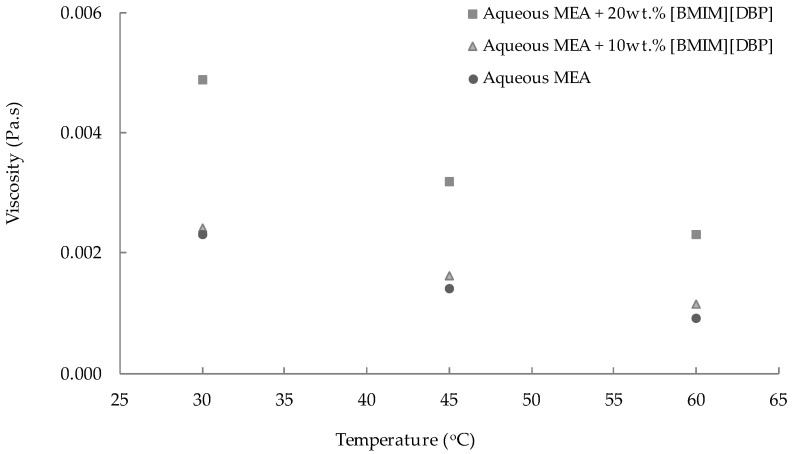
Viscosity of aqueous 30 wt%MEA-(BMIM)(DBP) hybrid solvent at different (BMIM)(DBP) concentrations, temperatures of 30 °C, 45 °C and 60 °C and a pressure of 1 atm.

The effect of IL concentration and temperature on the viscosity of the aqueous MEA-(BMIM)(DBP) hybrid solvent was studied, and results are shown in Figure 3. As shown in the figure, the viscosities of all absorbents used in this study were low (less than 0.05 Pa.s), which could help to facilitate the mass transfer for CO_2_ absorption [22]. The viscosity values of the aqueous 30 wt% MEA are in good agreement with those reported by Yang et al. [6]. This suggested that the viscosity data measured in this work were dependable. Figure 3 also demonstrates that the viscosity of the hybrid solvent decreased with increasing temperature. This is due to the weakening of the molecular resistance to flow when the temperature increased [24]. Meanwhile, an increase in the concentration of (BMIM)(DBP) increases the internal resistance in the mixture, which in turn increases the viscosity of the hybrid solvent. A similar observation was reported by Xu et al., Khan et al. and Zainul Anuar et al., where the viscosity of the hybrid solvent of aqueous MEA and IL increased greatly when the concentration of IL increased [22,24,25].

The density and viscosity data in Figure 2 and Figure 3 could be fitted using Equations (5) and (6).

(5) $$\rho =A_{1}+A_{2}T$$

(6) $$\ln\;\eta =A_{3}+\frac{A_{4}}{T}$$

where ρ is the density in g/cm^3^ and η is the viscosity in Pa.s of the aqueous 30 wt% MEA-(BMIM)(DBP) hybrid solvent. T is the temperature in K, and A_1_ through A_4_ are correlation coefficients using the least square method. The values of A_1_–A_4_ and the average absolute deviation (AAD) between the experimental and the calculated values from Equations (5) and (6) for the hybrid solvents are presented in Table 2 and Table 3, respectively. These correlations can be further employed to estimate the density and viscosity values at different temperatures.

### 3.2. Heat Capacity

**Figure 4:**
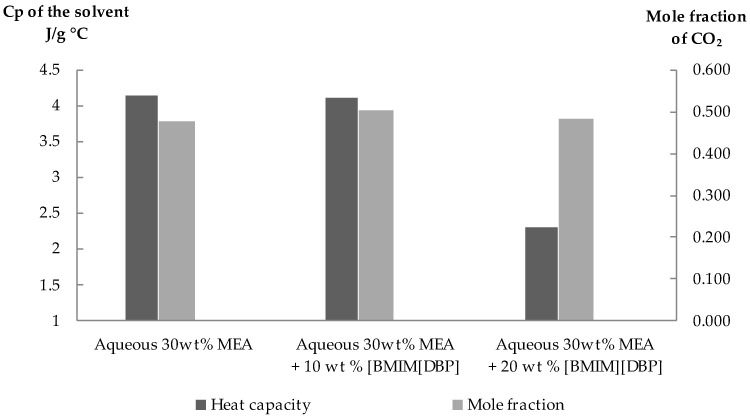
Heat capacity and CO_2_ absorption of MEA-(BMIM)(DBP) hybrid solvent. CO_2_ absorption measurement was carried out at 30 °C and 30 bar.

As shown in Figure 4, the heat capacity of the aqueous 30 wt% MEA-(BMIM)(DBP) hybrid solvent decreases with an increasing (BMIM)(DBP) concentration. The lower heat capacity of the hybrid solvents would save the energy during the heat-induced desorption of the CO_2_ for solvent regeneration purposes [26]. Therefore, this hybrid solvent mixture is potentially suitable for CO_2_ absorption. Yang et al. reported their simulation work on energy consumption for a mixture of aqueous 30 wt% MEA–30 wt% (BMIM)(BF_4_) and they found that the thermal energy for the mixed MEA-IL was 37.2% lower than that for the aqueous 30 wt% MEA solution [6].

It was also found that the solubility of CO_2_ in the hybrid solvent measured at a temperature of 30 °C and a pressure of 30 bar slightly decreased with increasing of the (BMIM)(DBP) concentration. A similar trend was reported for other hybrid solvents; MEA-(TBP)(MeSO_3_) by Zainul Anuar et al., MEA-(C_2_OHmim)(DCA) and MEA-(BMIM)(DCA) by Xu et al., MDEA-(N_11_)(Gly) by Feng et al. and MDEA-PZ-(BMIM)(OTf) by Khan et al. [8,22,25,27].

To evaluate the combined effect of (BMIM)(DBP) concentration with pressure and temperature on CO_2_ absorption in the aqueous 30 wt% MEA-(BMIM)(DBP) hybrid solvent, statistical analysis using RSM analysis was carried out to model and optimize the factors affecting CO_2_ absorption. Analysis of the heat capacity of the CO_2_-loaded sample of the hybrid solvent has also been a subject of interest. However, this work mainly focusses on the CO_2_ absorption of this hybrid solvent, and further studies on desorption for the regeneration process will be our next focus in the future.

### 3.3. CO_2_ Solubility in the Aqueous MEA-(BMIM)(DBP) Solvent Using RSM

#### 3.3.1. Data Collection and Fit Summary Analysis

In this study, the RSM face-centered CCD (FCCCD) method was employed to study the interaction of the factors towards the solubility of CO_2_ in aqueous 30 wt% MEA-(BMIM)(DBP) hybrid solvents. Three factors, namely, the amount of (BMIM)(DBP), temperature and pressure, were investigated. The experiments were conducted according to the design matrix generated by RSM. The observed experimental response data of the mole fraction of CO_2_ in the hybrid solvent (*Y*) are tabulated in Table 4. Table 5 provides the model summary statistics for CO_2_ absorption in aqueous 30 wt% MEA-(BMIM)(DBP). The model proposed by the software was the quadratic model, which is not aliased and is adequately significant to represent the correlation between CO_2_ absorption and operative parameters. Therefore, the quadratic model was selected for model fitting.

The final empirical model in terms of the coded factor for CO_2_ solubility (*Y*, mole fraction) is shown in Equation (7).

*Y* = 0.4927 − 0.0009*X*_A_ − 0.0356*X*_B_ + 0.1076*X*_C_ + 0.0115*X*_A_*X*_B_ + 0.0058*X*_A_*X*_C_ + 0.0058*X*_B_*X*_C_ + 0.0021*X*_A_^2^ − 0.0354*X*_B_^2^ + 0.0066*X*_C_^2^ (7)

where *Y* is the CO_2_ solubility (mole fraction), *X*_A_ is the (BMIM)(DBP) concentration (wt%), *X*_B_ is the temperature (°C) and *X*_C_ is the pressure (bar).

The coded factors in the quadratic model were beneficial for forecasting the relative significance of the factors by comparing the coefficient of the factors. Given the proposed correlation shown in Equation (7), the relative significance of the independent factors are as follows: *X*_A_ with a value of –0.0009, *X*_B_ with a value of –0.0356 and *X*_C_ with a value of +0.1076. The maximum increasing impact of the dependent factor was +0.0115, which is related to the interaction between *X*_A_ and *X*_B_ variable factors.

**Figure 5:**
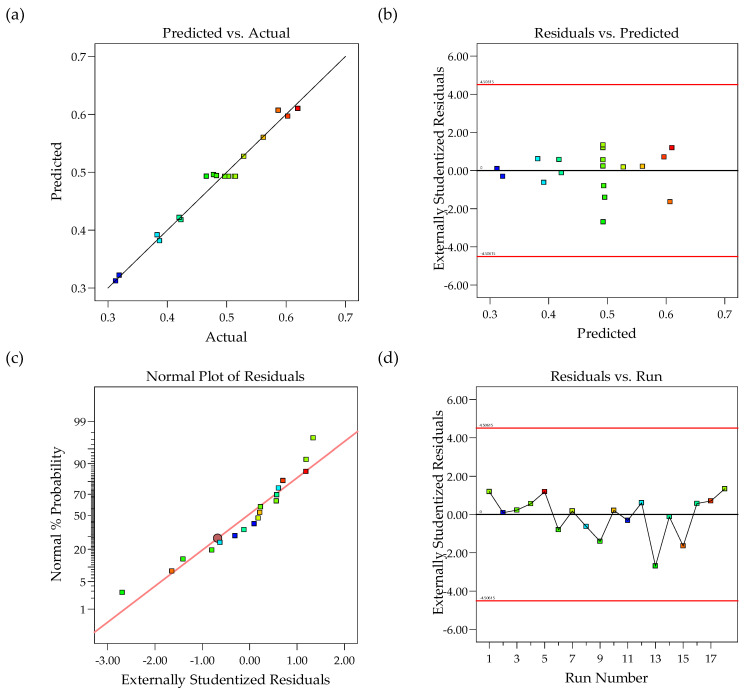
The CCD predicted value of CO_2_ removal efficiency vs. (**a**) actual absorption and (**b**) externally studentized residuals, and externally studentized residuals vs. (**c**) normal probability and (**d**) experiment run number.

Model fitness analysis was carried out by applying a lack of fit and analysis of variance (ANOVA) test. As can be seen from the analysis of variance (ANOVA) in Table 6, the calculated *p*-value of <0.0001 showed that the quadratic model was statistically significant (*p*-value < 0.05) with a low probability of error. The high value of *R*^2^ (0.979) indicates that the data fit the model very well. The adjusted *R*^2^ (0.9556) was in good agreement with the predicted value (0.9049), as the difference was less than 0.2. The adequate precision measures the signal-to-noise ratio, and the ratio value of 21.098 indicated an adequate signal, as an adequate precision >4 is favorable [17]. The “lack of fit” *F*-value of 7.38 implies that the “lack of fit” is not significant relative to the pure error. The non-significant (*p*-value = 0.0654) lack of fit can therefore be used with a low probability of error to navigate the design space [28,29]. Additionally, the low coefficient of variation (C.V.% = 3.96) and the good agreement of the observed vs. predicted CO_2_ solubility values (Figure 5a) suggest that the experiment was reliable.

Figure 5a shows a plot of the actual value against the predicted value for CO_2_ solubility, in which the points were randomly placed on a straight line. The experimental values and predicted values for all responses were close to each other, as shown in Table 4. These results confirmed that the predicted and actual values were in good agreement, with high acceptability of the models, and they can be applied to the analysis and prediction of CO_2_ absorption [30]. Figure 5b shows the random distribution of points up and down the *x*-axis inside the red line without any trends. This scenario suggested that the proposed models were acceptably free from any violation of the independence or constant variance assumption [31]. A normal probability chart of the studentized residuals is shown in Figure 5c as an additional tool to check the adequacy of the final model. This plot demonstrates an analysis of the normal probability plot of residuals that provides additional information on the adequacy of the final model. The graph shows an approximately linear residual distribution, which indicates a uniform distribution of errors, and shows that the model is sufficient [17]. Meanwhile, Figure 5d shows the residual plots vs. experimental results with randomly scattered points. The lack of an apparent trend in the plot indicates the absence of lurking variables that may have influenced the response during the experiment [32].

#### 3.3.2. Validation of Empirical Model Adequacy

To ensure the developed empirical model was accurate, three validation experiments were implemented with new parameters, which were not tested during the model development but were within the ranges used in the model. The tested operating conditions with their respective results are shown in Table 7. The experimental values agreed with predicted values estimated by RSM within a 95% confidence interval, indicating that the validity of the developed model is confirmed [33].

#### 3.3.3. Effect of Variable Factors in CO_2_ Solubility

**Figure 6:**
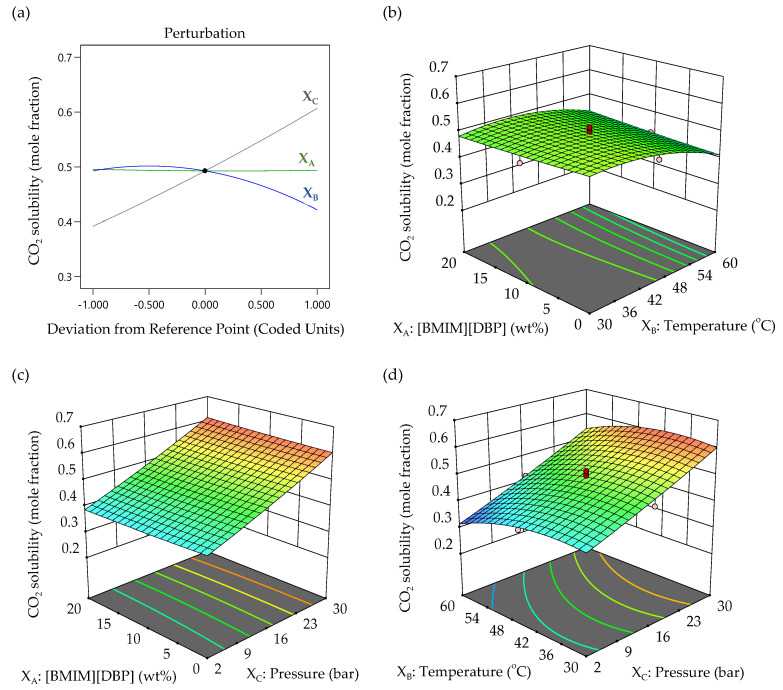
(**a**) Perturbation plot showing the effect of variable factors on CO_2_ solubility and the response surface plots of CO_2_ solubility as a function of (**b**) (BMIM)(DBP) concentration and temperature, at a pressure of 16 bar (**c**) (BMIM)(DBP) concentration and pressure at a temperature of 45 °C, and (**d**) temperature and pressure at a 10 wt% (BMIM)(DBP) concentration.

In this work, RSM was used to study the individual and interaction effects of the three independent factors on CO_2_ solubility in aqueous 30 wt% MEA-(BMIM)(DBP) for CO_2_ capture. A perturbation plot to compare the effect of all three factors is demonstrated in Figure 6a. As seen in the plot, the solubility of CO_2_ in aqueous 30 wt% MEA-(BMIM)(DBP) was not affected much by the (BMIM)(DBP) concentration (*X*_A_), decreased with increasing temperature (*X*_B_) and increased with increasing pressure (*X*_C_). This is in correlation with the ANOVA analysis result shown in Table 6, where the pressure (*X*_C_) factor was found to have the main impact on CO_2_ solubility. This finding is illustrated by the high *F*-value of 322.61 for pressure. The effects of IL concentration, temperature and pressure variables on the CO_2_ solubility mole fraction were further analyzed using simulated surface plots, according to the quadratic model.

The surface plots for the results achieved are shown in Figure 6b–d, where the data demonstrate the combined effect of the factors. The RSM surface plots are also useful to locate the best level of each factor for maximum CO_2_ absorption. Figure 6b shows the surface plot for the CO_2_ absorption as a function of the (BMIM)(DBP) concentration (*X*_A_) and temperature (*X*_B_) at a constant pressure of 16 bar. This plot demonstrates that increasing temperature decreased CO_2_ solubility, whereas increasing the IL concentration does not significantly affect the CO_2_ solubility. It was suggested by Xu et al. that the absorption of CO_2_ in aqueous MEA + ILs mainly relies on MEA but very little on ILs [22]. They also describe the ‘salting-out effect’, where adding salt into aqueous MEA reduces the solubility of carbamate in the solution. The same behavior of decreasing CO_2_ solubility with an increasing IL concentration was reported in previous publications [25,34]. Despite the reduced CO_2_ solubility from the effect of reduced water which was replaced by ILs, the presence of IL may help in saving energy during the regeneration process due to its lower heat capacity as compared to the heat capacity of water [6,22]. As shown above in Section 2.3, the mixture of aqueous MEA with 20 wt% (BMIM)(DBP) has lower heat capacity as compared to aqueous MEA and aqueous MEA with 10 wt% (BMIM)(DBP).

Figure 6c shows the CO_2_ solubility as a function of the (BMIM)(DBP) concentration (*X*_A_) and pressure (*X*_C_) at a constant temperature. By keeping the temperature constant at 45 °C, the maximum CO_2_ solubility showed a positive correlation with the pressure but was constant in relation to the IL concentration. The increase in CO_2_ solubility with increasing pressure at any given (BMIM)(DBP) concentration and temperature (as shown in Figure 6d) shows the direct effect of pressure. It was explained that as the pressure increases, the diffusion of CO_2_ into the solution increases; hence, more gas is dissolved [27]. On the other hand, the surface plot in Figure 6d also demonstrates that the CO_2_ solubility in the aqueous 30 wt% MEA-(BMIM)(DBP) slightly increased when the temperature (*X*_B_) increased at lower temperature and decreased with continual increases in temperature. The same trend of CO_2_ solubility has been reported previously, whereby the CO_2_ solubility increases with increasing pressure and decreases with increasing temperature in mixtures of aqueous amines and ILs, as well as in neat ILs [34,35].

#### 3.3.4. Optimized Simulation of the CO_2_ Absorption

Using the model developed using the RSM, it is possible to optimize the CO_2_ solubility in the aqueous 30 wt% MEA-(BMIM)(DBP). Optimization in RSM was carried out using a numerical optimization method. Based on the suggested RSM model, the optimum operating conditions were a 20 wt% (BMIM)(DBP) concentration, a temperature of 41.1 °C and a pressure of 30 bar. The predicted CO_2_ solubility at this condition was 0.617 mole fraction. The experimental value of a 0.599 mole fraction of CO_2_ was in agreement with the predicted value estimated by RSM within a 95% confidence interval. It was also found that the CO_2_ solubility in the aqueous 30 wt% MEA-(BMIM)(DBP) under optimum conditions was slightly lower than the solubility of CO_2_ in another reported hybrid solvent, i.e., MEA-(TBP)(MeSO_3_) hybrid solvent [25].

## 4. Conclusions

The solubility of CO_2_ in aqueous 30 wt% MEA-(BMIM)(DBP) was investigated using RSM. A quadratic model was proposed to correlate the factors and it was found that the model was able to predict the experimental data with an accuracy of R^2^ of 0.9791. The results showed that CO_2_ solubility increased with the increase of pressure and decreased with the temperature. Meanwhile, the concentration of (BMIM)(DBP) was found not to significantly affect the CO_2_ absorption but it was also found that with the addition of (BMIM)(DBP), the heat capacity of the MEA-(BMIM)(DBP) solvent is lower; hence, it might reduce the energy needed during the regeneration process. The optimum conditions to maximize the CO_2_ absorption in aqueous 30 wt% MEA-(BMIM)(DBP) were obtained at 20 wt% (BMIM)(DBP), a temperature of 41.1 °C, and a pressure of 30 bar. The results of validation runs showed that the experimental values agreed with predicted values estimated by RSM within a 95% confidence interval.

## Figures and Tables

**Table 1:** Independent variables and concentration levels for response surface studies.

Factors	Unit	Levels		
		−1	0	+1
*X*_A_: (BMIM)(DBP) concentration	wt%	0	11	20
*X*_B_: Temperature	°C	30	45	60
*X*_C_: Pressure	bar	2	16	30

**Table 2:** Fitting parameters of Equation (5) to correlate density (ρ) of aqueous 30 wt% MEA-(BMIM)(DBP).

Solvent	A_1_	A_2_	100AAD
Aqueous 30 wt%MEA-10 wt% (BMIM)(DBP)	1.1940	−0.0005	0.02
Aqueous 30 wt%MEA-20 wt% (BMIM)(DBP)	1.2228	−0.0006	0.02

**Table 3:** Fitting parameters of Equation (6) to correlate viscosity (η) of aqueous 30 wt% MEA-(BMIM)(DBP).

Solvent	A_3_	A_4_	100AAD
Aqueous 30 wt%MEA-10 wt% (BMIM)(DBP)	−7.3863	2.5059	0.52
Aqueous 30 wt%MEA-20 wt% (BMIM)(DBP)	−6.7494	2.5235	1.29

**Table 4:** The face-centered central composite design (FCCCD) design matrix and the mole fraction of CO_2_ in the aqueous 30 wt%MEA-(BMIM)(DBP) hybrid solvents.

Standard	*X*_A_: IL Concentration (wt%)	*X*_B_: Temperature (°C)	*X*_C_: CO_2_ Pressure (bar)	*Y*: Mole Fraction Experimental	*Y*: Mole Fraction
					Predicted
1	0	30	2	0.423	0.418
2	20	30	2	0.387	0.382
3	0	60	2	0.313	0.312
4	20	60	2	0.319	0.322
5	0	30	30	0.620	0.610
6	20	30	30	0.603	0.597
7	0	60	30	0.529	0.527
8	20	60	30	0.562	0.560
9	0	45	16	0.478	0.496
10	20	45	16	0.483	0.494
11	10	30	16	0.466	0.493
12	10	60	16	0.420	0.422
13	10	45	2	0.383	0.392
14	10	45	30	0.587	0.607
15	10	45	16	0.503	0.493
16	10	45	16	0.513	0.493
17	10	45	16	0.497	0.493
18	10	45	16	0.515	0.493

**Table 5:** Fit summary output analysis.

Source	Standard Deviation	*R* ^2^	Adjusted *R*^2^	Predicted *R*^2^	Remarks
Linear	0.0251	0.9358	0.922	0.9009	
2FI	0.0256	0.9473	0.9186	0.8737	
Quadratic	0.0189	0.9791	0.9556	0.9049	Suggested
Cubic	0.0243	0.9828	0.927	−20.9735	Aliased

**Table 6:** ANOVA for quadratic modeling of CO_2_ absorption.

Source	Sum of Squares	dF	Mean Square	*F*-Value	*p*-Value	
Model	0.1344	9	0.0149	41.61	<0.0001	Significant
*X*_A_-IL Concentration	8.10 × 10^−6^	1	8.10 × 10^−6^	0.0226	0.8843	
*X*_B_-Temperature	0.0127	1	0.0127	35.31	0.0003	
*X*_C_-Pressure	0.1158	1	0.1158	322.61	<0.0001	
*X*_A_ *X*_B_	0.0011	1	0.0011	2.95	0.1243	
*X*_A_ *X*_C_	0.0003	1	0.0003	0.737	0.4156	
*X*_B_ *X*_C_	0.0003	1	0.0003	0.737	0.4156	
*X*_A_^2^	0	1	0	0.0324	0.8616	
*X*_B_^2^	0.0034	1	0.0034	9.48	0.0152	
*X*_C_^2^	0.0001	1	0.0001	0.3261	0.5837	
Residual	0.0029	8	0.0004			
Lack of Fit	0.0027	5	0.0005	7.38	0.0654	Not significant
Pure Error	0.0002	3	0.0001			
Cor Total	0.1373	17				
Std. dev	0.0189		C.V.%	3.96		
Mean	0.4778		Adeq precision	21.098		

**Table 7:** Results of operating conditions with experimental design in confirmation runs.

Run Factor			CO_2_ Solubility (Mole Fraction)		
*X*_A_: IL Concentration (wt%)	*X*_B_: Temperature (°C)	*X*_C_: Pressure (Bar)	Experimental Value	Lower Limit	Higher Limit
30	30	30	0.639	0.544	0.689
20	30	16	0.489	0.425	0.489
10	30	02	0.402	0.340	0.402

## Data Availability

Not applicable.
